# An elevated lipoprotein (a) level, rapid progression, and accumulation of lipidic plaque contents in a deferred coronary artery lesion despite lipid-lowering therapy

**DOI:** 10.1093/ehjcr/ytaf255

**Published:** 2025-05-19

**Authors:** Tomomi Watanabe, Yu Kataoka, Aya Katasako, Teruo Noguchi

**Affiliations:** Department of Cardiovascular Medicine, National Cerebral and Cardiovascular Center, 6-1 Kishibe-shinmachi, Suita, Osaka 564-8565, Japan; Department of Cardiovascular Medicine, National Cerebral and Cardiovascular Center, 6-1 Kishibe-shinmachi, Suita, Osaka 564-8565, Japan; Department of Cardiovascular Medicine, National Cerebral and Cardiovascular Center, 6-1 Kishibe-shinmachi, Suita, Osaka 564-8565, Japan; Department of Cardiovascular Medicine, National Cerebral and Cardiovascular Center, 6-1 Kishibe-shinmachi, Suita, Osaka 564-8565, Japan

## Case description

**Figure 1:**
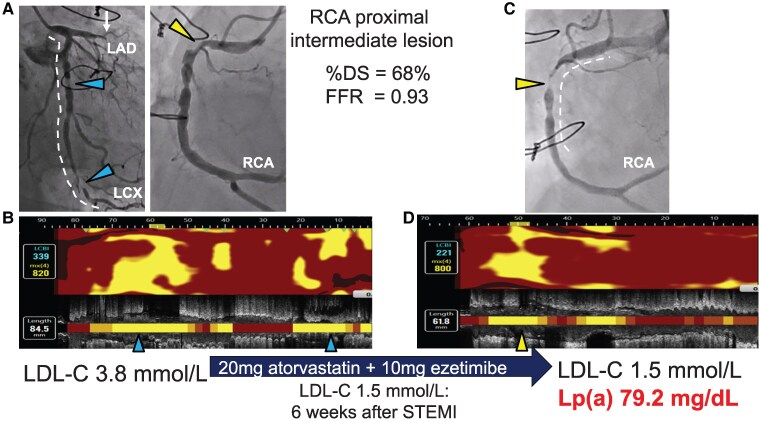
Rapid disease progression at deferred coronary artery lesions. Coronary angiography on 1st admission due to ST-segment-elevation myocardial infarction. The left anterior descending artery was occluded (white arrow). Two severe stenoses (blue triangles) in the left circumflex artery and one intermediate stenosis (yellow triangle) in the right coronary artery are also observed. Fractional flow reserve for the right coronary artery was 0.93. The white dotted line represents the image segment obtained using Near-infrared spectroscopy. The blue triangles correspond to those in (*B*). (*A*) Near-infrared spectroscopy chemogram within the left circumflex artery prior to percutaneous coronary intervention. Extensive lipid materials were present with a maxLCBI_4mm_ of 820. The LDL-cholesterol level was 3.9 mmol/L. Following the completion of percutaneous coronary intervention, 20 mg atorvastatin and 10 mg ezetimibe were initiated, and the LDL-cholesterol level decreased to 1.6 mmol/L (58% reduction). (*C*) Coronary angiography at 6 months post-percutaneous coronary intervention. The deferred lesion in the right coronary artery has rapidly progressed (yellow triangle) and caused NSTEMI. The white dotted line represents the image segment obtained using Near-infrared spectroscopy. The yellow triangle corresponds to that shown in (*D*). (*D*) Near-infrared spectroscopy chemogram within the right coronary artery prior to the percutaneous coronary intervention. Despite lipid-lowering therapy (LDL-cholesterol, 1.5 mmol/L), there were substantial lipidic plaque materials in theright coronary artery. In addition, the Lp(a) level was 79.2 mg/dL. FFR, fractional flow reserve, LAD, left anterior descending artery, LCX, left circumflex artery, LDL-C, low-density lipoprotein cholesterol, Lp(a), lipoprotein (a), NSTEMI, non-ST-segment elevation myocardial infarction, PCI, percutaneous coronary intervention, RCA, right coronary artery, STEMI, ST-segment elevation myocardial infarction.

A 79-year-old male was hospitalized due to an abnormal ECG and akinesis in the anterior region on echocardiography. He had prolonged chest pain 2 weeks prior to hospitalization, which suggested ST-segment-elevation myocardial infarction (STEMI) 2 weeks previously. Coronary angiography revealed occlusion of left anterior descending, accompanied by two severe stenoses of left circumflex (LCX) artery and an intermediate stenosis at the proximal segment of right coronary artery (RCA) (see Supplementary material 8 online, *Movies SI* and *SII*). ECG and echocardiography suggested a non-viable myocardium in the anterior region, and wire-based fractional flow reserve (FFR) (Optowire, Zeon Medical, Japan) for an intermediate stenosis of the RCA was 0.93. Any intravascular imaging was not conducted to evaluate plaque features at this intermediate stenosis. Therefore, percutaneous coronary intervention (PCI) was performed on the LCX artery. Near-infrared spectroscopy (NIRS) imaging (Dualpro^TM^, Infraredx, Bedford, MA, USA) revealed extensive lipidic plaque materials [maximum lipid-core burden index (LCBI_4mm_) = 820] (*Figure 1*, Supplementary material 8 online, *Movie SIII*). Following completion of PCI, 20 mg atorvastatin and 10 mg ezetimibe were initiated, and LDL-cholesterol (LDL-C) level decreased to 1.6 mmol/L (58% reduction). Lipoprotein (a) (Lp[a]) level was not measured during initial hospitalization, whereas on-treatment LDL-C level was 1.5 mmol/L, 6 weeks post-hospitalization. However, he was re-hospitalized due to NSTEMI 6 months post-PCI (on-treatment LDL-C = 1.5 mmol/L). Coronary angiography revealed rapid progression of non-critical stenosis at the proximal segment of the RCA, which had an FFR ≥0.91 6 months previously (*Figure 1*, Supplementary material 8 online, *Movies SIV* and *SV*). NIRS imaging revealed substantial lipidic plaque materials at this lesion despite achieving LDL-C < 1.8 mmol/L (maxLCBI_4mm_ = 800) (*Figure 1*, Supplementary material 8 online, *Movie SVI*). Furthermore, he had an elevated Lp(a) level (79.2 mg/dL).

Recent observational studies reported very low event rates at lesions with an FFR ≥0.91.^1^ In our case, despite non-critical feature of stenosis at the proximal segment of the RCA, an accelerated progression with ongoing accumulation of lipid was observed during lipid-lowering therapy. Given the pro-atherogenic properties of Lp(a),^2^ the residual elevated Lp(a) level may require additional novel therapies for STEMI.

## Supplementary Material

ytaf255_Supplementary_Data

## Data Availability

The data underlying this article will be shared upon reasonable request to the corresponding author.
