# Supplementary material for: An elevated lipoprotein (a) level, rapid progression, and accumulation of lipidic plaque contents in a deferred coronary artery lesion despite lipid-lowering therapy
Source: Eur Heart J Case Rep. 2025 May 19;9(5):ytaf255. doi: 10.1093/ehjcr/ytaf255 (PMC12120835; doi:10.1093/ehjcr/ytaf255)
Supplement: ytaf255_Supplementary_Data [file ytaf255_supplementary_data.zip › Supplementary Movie Legends.docx]

**Supplementary Movie Legends**

**Movie I. Left coronary angiography**

The proximal segment of LAD was occluded. There were two severe stenosis in his LCX.

**Movie II. Right coronary angiography**

One intermediate stenosis at the proximal of RCA was identified.

**Movie III. NIRS/IVUS imaging prior to PCI for his LCX**

Extensive lipidic materials existed within the entire segment of LCX.

**Movie IV. Left coronary angiography 6 months after PCI**

There were no disease progression and in-stent restenosis in his LCA.

**Movie V. Right coronary angiography 6 months after PCI**

Rapid progression of deferred coronary lesion was observed.

**Movie VI. NIRS/IVUS imaging prior to PCI for his RCA**

Despite achieving LDL-C <1.8mg/dL, substantial amount of lipidic plaque materials existed at the deferred lesion (maxLCBI_4mm_=800).

IVUS = intravascular ultrasound, LAD = left anterior descending artery, LCA = left coronary artery, LCX = left circumflex artery, LDL-C = low-density lipoprotein cholesterol, NIRS = near-infrared spectroscopy, PCI = percutaneous coronary intervention, RCA = right coronary artery
